# Seeking Healthcare During Lockdown: Challenges, Opportunities and Lessons for the Future

**DOI:** 10.34172/ijhpm.2021.26

**Published:** 2021-04-13

**Authors:** Fiona Imlach, Eileen McKinlay, Jonathan Kennedy, Megan Pledger, Lesley Middleton, Jacqueline Cumming, Karen McBride-Henry

**Affiliations:** ^1^Health Services Research Centre, Victoria University of Wellington, Wellington, New Zealand.; ^2^Department of Primary Health Care and General Practice, University of Otago, Wellington, New Zealand.; ^3^School of Nursing, Midwifery and Health Practice, Victoria University of Wellington, Wellington, New Zealand.

**Keywords:** Primary Healthcare, Pandemic, COVID-19, Access, Health Services, New Zealand

## Abstract

**Background:** In Aotearoa/New Zealand, the first nation-wide coronavirus disease 2019 (COVID-19) lockdown occurred from March 23, 2020 to May 13, 2020, requiring most people to stay at home. Health services had to suddenly change how they delivered healthcare and some services were limited or postponed. This study investigated access to healthcare during this lockdown period, whether patients delayed seeking healthcare and reasons for these delays, focusing on the accessibility of primary care services.

**Methods:** Adults (aged 18 years or older) who had contact with primary care services were invited through social media and email lists to participate in an online survey (n = 1010) and 38 people were recruited for in-depth interviews. We thematically analysed qualitative data from the survey and interviews, reported alongside relevant descriptive survey results.

**Results:** More than half (55%) of survey respondents delayed seeking healthcare during lockdown. Factors at a national or health system-level that could influence delay were changing public service messages, an excessive focus on COVID-19 and urgent issues, and poor service integration. Influential factors at a primary care-level were communication and outreach, use of technology, gatekeeping, staff manner and the safety of the clinical practice environment. Factors that influenced patients’ individual decisions to seek healthcare were the ability to self-manage and self-triage, consciousness of perceived pressure on health services and fear of infection.

**Conclusion:** In future pandemic lockdowns or crises, appropriate access to primary care services can be improved by unambiguous national messages and better integration of services. Primary care practices should adopt rapid proactive outreach to patients, fostering a calm but safe clinical practice environment. More support for patients to self-manage and self-triage appropriately could benefit over-burdened health systems during lockdowns and as part of business as usual in less extraordinary times.

## Background

**Key Messages**

**Implications for policy makers**

- Those who delayed seeking care were more likely to be struggling financially, be concerned about the impact of coronavirus disease 2019 (COVID-19), have poorer health, a disability or more difficulties in managing their health in the pandemic.
- Individuals considered the risk of COVID-19 exposure to themselves and others and managed non-urgent problems by watching and waiting and self-care.
- Proactive, up-to-date communication from practices about what services were available, how to get in touch and expected changes to service delivery could encourage access to services but needed to reach those most at risk.
- Practices with systems already in place to undertake electronic, patient-focused communication were better able to provide care during lockdown.
- Outreach by non-digital methods can ensure that patients without online capability are not excluded.

**Implications for the public**

This study offers insights into how people experienced accessing healthcare during the pandemic. It highlights that people did delay, or did not, access healthcare if they believed their need for healthcare was not urgent, or if the risk of being coming ill because of the pandemic was deemed too great. The study also reflects the people’s views on how primary care should operate during a pandemic; this information can support future health service planning.

The novel coronavirus disease 2019 (COVID-19) has spread rapidly throughout the globe since its emergence in December 2019, infecting millions of people and causing over a million deaths as of September 30, 2020.^1^ Different countries have taken a range of approaches to constrain the impact of the virus, including intensive community testing and tracing of contacts, travel restrictions, mask-wearing and partial to complete ‘lockdowns,’ where all non-essential workers are instructed to stay at home.^2^

 Aotearoa/New Zealand responded to the COVID-19 threat with a nationwide lockdown from March 23, 2020, until May 13, 2020. During the complete lockdown period, from March 25 to April 27, 2020 (level four of a four-level alert level system introduced to manage the pandemic response),^3^ only essential services remained open (including supermarkets, pharmacies, general practices and public hospitals), with much routine healthcare put on hold, and many consultations performed remotely by telephone or video (telehealth). People were expected to keep within their neighbourhoods, limit contact to household members, and maintain physical distance from others. During level three, the partial lockdown period that ended on May 13, 2020, early childhood centres, schools and takeaway food services could open with restrictions. Health services were still advised to operate remotely as much as possible, to avoid them becoming the focus of virus spread, as had happened elsewhere.^4^

 During lockdown, televised daily briefings from the Prime Minister and Director-General of Health updated the nation on the number of new COVID-19 cases, hospitalisations and deaths, and provided messages about accessing healthcare and COVID-19 testing. These messages were repeated via regular advertising on television, social media and news channels. In the early stages of lockdown, there was a high level of concern that both primary and secondary healthcare services could be overwhelmed by the pandemic.^5^ However, it quickly became apparent that demand for primary care declined during lockdown (apart from an initial surge for repeat prescriptions and flu vaccinations as the winter flu session approached),^6^ with concomitant reduced hospital emergency department (ED) presentations and acute hospital admissions.^7,8^ This led to a new concern, that delays in seeking healthcare could lead to serious problems being missed or an unmanageable deluge of demand once lockdown ended.^6,7^

 Declines in healthcare utilisation during COVID-19 lockdowns have also been reported internationally.^9^ In the United Kingdom, general practitioner appointments in April-May 2020 were down by a third compared to the same period the year before.^10^ Cancer screening appointments decreased in the United States and cancer diagnoses decreased in the Netherlands.^11^ Delays in seeking paediatric healthcare contributed to avoidable harm and even death for children during the 2020 lockdown in Italy, attributed to parental fear of COVID-19.^12^ An inability to access care or delays in seeking care were likely explanations for a significant reduction in ED presentations for heart attacks and strokes in the United States during March to May 2020.^13^

 It is worth noting that prior to the 2020 lockdown, inequities were evident in relation to accessing primary healthcare within New Zealand,^14^ an issue that has been exacerbated by COVID-19.^9^ In particular, inequities related to access to primary healthcare existed for the indigenous population Māori,^15^ those with mental health challenges,^16^ multimorbidity^17^ and those with lower socioeconomic status^18^ prior to COVID-19 lockdown. There is little research being published about the impact of lockdown on access to primary healthcare for vulnerable groups in Aotearoa/New Zealand.

 This study aimed to obtain a patient perspective on accessing healthcare during the first COVID-19 lockdown in Aotearoa/New Zealand, with a particular focus on general practice, as the front-line health service for the pandemic.^19^ For the purposes of this research, ‘access’ to healthcare refers to health service utilisation and help seeking by the participants. Research questions included:

- What contact did patients have with health services during lockdown?
- Did patients delay seeking healthcare and for what reasons?
- What changes would improve access to general practice in a future crisis?

This is one of a series of papers about patient experiences of healthcare during lockdown; others report on telehealth^20^ and pharmacy/prescribing.^21^ This paper reports on barriers to healthcare during lockdown highlighting negative experiences, however in another analysis we found that those who accessed healthcare had predominantly positive experiences.^20^

 For context, the health system in Aotearoa/New Zealand is set up to provide free emergency and hospital services but general practices operate as private organisations, using a wide range of business models.^22^ Most of these involve patient co-payments for services, subsidised by a Government capitation fee, based on the characteristics of the patients enrolled in the general practice (higher capitation funding applies to practices that serve populations with higher health needs). Services are free for children less than 14 years old.

## Methods

 A mixed method approach was used, with an anonymous online survey of adults (18 years and older) who had, or wanted to have, contact with health services during lockdown, supplemented by semi-structured interviews. Given the constraints of lockdown, the survey and interview schedule were developed by experienced health researchers within the research team, informally reviewed by external experts, and consumer pilot testing was done with household contacts of the research team.

###  Online Survey

 The survey was online from April 20 to May 13, 2020 (within lockdown levels four and then level three) and asked about contact with health services and if people had delayed seeking healthcare during lockdown (questionnaire in Supplementary file 1). Eight possible reasons for delay were presented, based on previous surveys (eg, cost and transport)^23^ and likely reasons in the context of lockdown (eg, fear of infection, services unavailable). Socio-demographic questions included age, gender, ethnicity, postcode and work status. Health status was assessed with questions on self-rated health, disability status and existing long-term conditions. Other questions asked whether the pandemic made it easier or more difficult to manage health, and level of concern about the impact of the pandemic on health. Responses to open-ended questions were included in the qualitative data analysis.

 Recruitment was through snowballing using social media and email lists, sent through personal and professional networks, including the Universities affiliated with the research and other organisations. Only responses from those who lived in Aotearoa/New Zealand, who completed >70% of the survey and provided >20 responses were included in the final analysis (n = 1010). Descriptive survey statistics only are presented, with missing values dropped from the analysis, assuming that respondents with missing values were similar to those without. The sampling errors in a snowball sample are not meaningful so judgements have to be made pragmatically. However, as a guide, the maximum margin of error of a simple random sample of equivalent size to the groups we analyse here are 3.1% (n = 1010), 4.2% (n = 542) and 4.7% (n = 444).

###  Interviews

 Interviewees were recruited from the online survey. Four hundred and thirty-six survey respondents provided name and contact details (collected separately from survey responses) for follow-up. From these, we purposefully sampled by gender and invited 75 people for an in-depth interview, of whom 41 agreed to and 38 completed an interview within the project timeframe (others did not respond). Interviewees were sent information about the research, provided oral or written consent prior to the interview, and were given a gift-voucher for their time. Interviews were done via Zoom or telephone, audio-recorded, transcribed, checked and could be reviewed by the interviewee on request.

 Interviewees were asked in more detail about their experiences of accessing healthcare and reasons for delaying seeking care. Interviews were conducted by EM, FI, LR, MC, JK, JM and took on average 33 minutes.

###  Qualitative Data Analysis

 We used thematic analysis and a mixture of deductive and inductive coding to analyse the 38 interview transcripts and open-ended survey questions. The coding of the transcripts was managed using NVivo 11 Pro (QSR International). We applied Levesque’s framework of access to healthcare^24^ as an initial coding frame. This framework describes the intersection of patients’ abilities, and demand for services, with supply features of health services (all of which may be disrupted by lockdowns and pandemics). From this initial analysis, we developed more nuanced themes^25^ around what could enable or hinder access to healthcare. Themes from interviews were checked against the analysis of open-ended questions to confirm the completeness of the analysis. Quotes are inserted verbatim, with identifiers including age range, gender and whether from survey (S) or interview (I).

## Results

###  Sample Characteristics

 Characteristics of survey respondents and interviewees are shown in Supplementary file 2. There was a predominance of females and those from the lower North Island of Aotearoa/New Zealand. Interviewees were more likely to be older and not in employment than survey respondents.

###  Quantitative Results

####  Contact With Health Services

 Most respondents (86%) reported contact with general practice during lockdown, either for themselves or someone else. (More details about the types of contacts with general practices and experiences of telehealth are published elsewhere^20^). Contact with community pharmacy was also common (56%); 15% had contacted Healthline (free national telephone health information service); 11% had experience of a community-based COVID-19 testing centre and 8% had contacted another health professional (eg, mental health, physiotherapy, maternity care). Respondents also reported contact with a variety of other health services: 9% attended a hospital outpatient or specialist appointment; 6% attended a public hospital ED and another 6% visited a private after-hours clinic; 3% had imaging or lab testing; and another 3% had a hospital admission. (Multiple responses were possible; hence these totals add to more than 100%).

####  Delay in Seeking Healthcare

 Over half (55%) of survey respondents delayed seeking healthcare during lockdown, and were more likely to be younger, have a disability, have poorer self-rated health and struggle to pay for living costs, find it more difficult to manage their health during the pandemic and be more concerned about the impact of the pandemic on their health (see Table 1).

**Table 1:** Characteristics of People Who Delayed or Did Not Delay Seeking Healthcare

**Characteristics**	**Delayed Seeking Healthcare** ^a,b^ **(n = 542)** **No. (%)**	**Did Not Delay Seeking Care** ^a,b^ ** (n = 444)** **No. (%)**
Age		
18-34	139 (26)	79 (18)
35-44	120 (22)	78 (18)
45-54	139 (26)	105 (24)
55-64	82 (15)	89 (20)
65+	56 (10)	89 (20)
Gender		
Female	464 (86)	366 (83)
Male	68 (13)	71 (16)
Other	8 (1)	5 (1)
Prioritised ethnicity		
Māori	51 (10)	45 (10)
Pacific peoples	13 (2)	5 (1)
Asian	22 (4)	11 (3)
New Zealand European/other	448 (84)	379 (86)
Current work status		
In paid employment as before COVID-19	303 (57)	270 (61)
In paid employment with reduced pay	61 (11)	47 (11)
In paid employment not being paid	18 (3)	8 (2)
Unemployed looking for work	22 (4)	8 (2)
Not in paid employment, not looking for work	130 (24)	108 (24)
Struggle to pay for living costs in last 7 days		
Agree/strongly agree	57 (11)	16 (4)
Neither	68 (13)	34 (8)
Disagree/strongly disagree	414 (77)	391 (89)
Self-rated health		
Excellent	51 (9)	67 (15)
Very good	194 (36)	184 (42)
Good	176 (33)	144 (33)
Fair	93 (17)	44 (10)
Poor	26 (5)	4 (1)
Presence of long term health condition	336 (62)	268 (61)
Presence of disability	78 (14)	43 (10)
Concerned^c^ about impact of COVID-19 on their own health	284 (52)	184 (41)
More difficult^d^ to manage health during pandemic	306 (57)	88 (20)

Abbreviation: COVID-19, coronavirus disease 2019. ^a^ Missing values were excluded from the analyses. ^b^The maximum margin of error for a simple random samples of size 542 and 444 are 4.2% and 4.7%, respectively. ^c^ Extremely, very or moderately concerned (other question responses were slightly or not at all concerned). ^d^A little or much more difficult (other question responses were a little easier, much easier or neither).

 Most common reasons for delay were a concern that health services were busy (52%); that services were postponed or delayed (37%) or not available (27%); fear of being infected with COVID-19 (31%), or infecting others (8%). Cost (9%) and transport (4%) were less often reported as reasons for delay. Six percent of respondents specified in the open-ended response option that they delayed because their problem was not urgent enough, usually from their own judgment of what was urgent.

###  Qualitative Results

 Respondents described their experiences mainly in relation to general practice but also mentioned other health services. They reported influences on accessing health services at three levels: (1) National/health system; (2) General practice; and (3) Individual. These influences could either enable or hinder access across four of Levesque’s dimensions of access to healthcare^24^ (see Table 2): the patient’s ability to perceive their health need, aligned with the approachability of services; the ability to seek healthcare that was acceptable; the ability to reach services that were available and accommodating; and the ability to engage in appropriate healthcare services. We did not find influences for all dimensions and levels. Responses about ability to pay and affordability of services related almost exclusively to telehealth, which is discussed elsewhere.^20^

**Table 2:** Influences on Access to Primary Care During Lockdown by System, Practice and Individual-Level Factors

	**Influences on Access**		
**Dimensions of Access** ^24^	**National/Health System-Level **	**General Practice-Level **	**Individual-Level **
Ability to perceive/approachability	1.1 Public service messages	2.1 Communication and outreach	3.1 Self-management and self-triage
Ability to seek/acceptability		2.2 Use of technology	3.2 Conscious of health services pressure
Ability to reach/availability and accommodation	1.2 Focus on COVID-19 and urgent issues	2.3 Gatekeeping	3.3 OK/not OK to seek routine healthcare
Ability to engage/appropriateness	1.3 Service integration	2.4 Staff manner 2.5 Safety of the health service environment	3.3 Fear of infection

Abbreviation: COVID-19, coronavirus disease 2019.

####  1. National/Health System Influences on Healthcare Access

#### 1.1. Public Service Messages 

 During lockdown, people reported relying on national news outlets for health information, especially the televised daily updates. On the whole, people found these reassuring and informative. However, at the start of lockdown, people noted that messages emphasised being alert for signs of COVID-19, were focused on containing the virus and directed people to telephone Healthline. As time went on, it was recognised that people were not accessing care for non-COVID-19 conditions, and messages encouraged access to general practices for any health concerns. This change in messaging introduced some confusion about how to access healthcare services, and for what, and was perceived to conflict with the general stay-at-home advice. People were also unclear about whether they were allowed to travel for healthcare that was not local (eg, for those in rural settings or who attended a general practice outside of their home suburb).

*“I think there have been mixed messages about seeking GP help. Initially we were asked to stay away, only go in extreme circumstances, call the 0800 number … and then we’re told people aren’t going and to remember to go when you need help. For me it did delay in getting my … asthma dealt with” *(Survey response: Female, aged 45-54).

#### 1.2. Focus on COVID-19 and Urgent Issues

 In some instances, respondents felt their non-COVID-19 health issues were not taken seriously or given proper consideration, particularly at the start of lockdown when concerns about COVID-19 were dominant. Respondents reported staff spending more time on screening for COVID-19 symptoms than attending to their presenting complaint. People who had health issues that were not deemed sufficiently urgent were deferred or denied care, even if this could have been delivered through telehealth (eg, support for smoking cessation (S: F, 45-54)), or should have been a priority (eg, flu vaccinations (S: F, 35-44)). Elective surgery and specialist appointments were postponed or cancelled, sometimes without any indication about rescheduling. Concerns were also expressed about the long-term consequences of delaying healthcare for non-COVID-19 issues (eg, cancer screening), and missing out on imaging and laboratory testing.

*“The lockdown meant I couldn’t get see my GP for examination, couldn’t get a mammogram or ultrasound for a breast lump for 6 weeks even though I am [in] a high-risk group… It put my health at risk when the testing could have been managed safely”* (S: F, 45-54).

 People with respiratory symptoms from pre-existing problems sometimes had care delayed until a COVID-19 test was done. Lab tests were sometimes unavailable or unable to be processed due to overload from COVID-19 testing.

#### 1.3. Service Integration

 Although people were usually positive about their interactions with individual service providers, integration of services between general practice and other organisations was variable. A commonly reported example was the interface between general practices and pharmacies. Although the Ministry of Health quickly enabled more widespread electronic prescribing,^26^ this took time for some practices and pharmacies to adopt effectively, and led to delays in patients getting medicines they needed, particularly early in lockdown. Similarly, poor co-ordination between Healthline and other health services could cause difficulties in arranging COVID-19 tests or getting advice for non-COVID-19 issues. The sense of being bounced from one service to another and getting inadequate help was compounded by long wait times or an inability to get through on the phone.

*“The different departments gave different advice on whether I should take my elderly relative for urgent assessment … I rang the ED who said I should ring Healthline, Healthline said I should ring GP. GP had said I should take them to ED. When we got to ED I was unable to advocate for my relative [due to visitor restrictions], so the diagnosis appeared to neglect several aspects of medical history that had been forgotten by my relative. Five days later treatment has been ineffective, and I am left in limbo as to where to go”* (S: F, 65-74).

 For some patients who required support from multiple agencies, including provision of care in the home, the terminally ill, and those waiting for specialist review or recovering post-surgery, inadequate care coordination and having no-one taking the lead for their care during lockdown caused more distress.

*“[My sister] got a phone call from the oncologist on the Friday which would have been about the 27th of March to say that no, there was no more treatment they could offer her, no more chemo [because the cancer was too extensive] … They had to chase everyone up. Nobody contacted them … My sister felt abandoned. That’s her words, totally abandoned” *(Interview response: F, 45-54).

####  2. General Practice Influences on Healthcare Access 

#### 2.1. Communication and Outreach

 Communication between practices and patients was perceived to vary from excellent to poor. It ranged from general practice staff in a deprived area standing outside the practice building in personal protective equipment (PPE) to assure those driving past that health services were available; to practices that sent messages by email, text or through patient portals; through to practices that sent no information to patients about how to access care.

*“I honestly would have really appreciated an email or something; some sort of contact about what my GP clinic was doing or expecting from their patients” *(I: F, 18-24).

 Respondents valued up-to-date websites and social media platforms such as Facebook. These, coupled with voice messages on practice telephones, were often the first line of information about whether/when the practice was open and for what, and needed to be updated quickly. This was especially important early on, when people received a deluge of emails from different companies and groups, leading to ‘alert fatigue’ (a phrase coined by one respondent (I: M, 25-34)).

 Proactive outreach from practices was highly valued but far from universal. One respondent reported how this worked well, receiving an email from her practice to warn her of being at ‘high risk’ of complications from COVID-19, which was then followed up by phone to make plans to prevent infection (S: F, 18-24). Another reported how practices were ringing patients to check on their welfare and make sure they had enough medication and reflected on how that felt: ‘they took the time to call me, they reached into my world instead of me reaching into theirs and running a gauntlet to get the information’ (I: F, 45-54).

#### 2.2. Use of Technology

 Usual methods of contacting general practices changed during lockdown. Respondents praised practices that made use of existing or new digital technologies from the start of lockdown or soon after, both for communication and service delivery. Patient portals and email communication were popular as they greatly enhanced access to care in the lockdown context, but ineffective use of these tools created barriers, particularly when useful functions were disabled or ignored. For example, general practices that withdrew the option for online appointment bookings to prioritise triaging of all appointments by telephone caused frustration with long waiting times to get through on the phone. Practices that already had well set-up systems were able to quickly provide services appropriate for lockdown such as telehealth and online prescriptions whereas practices without pre-existing systems did not function as well.

*“I’ve been booking appointments and asking for repeats through [the online portal] for a long time but now … I can actually just email my doctor” *(I: F, 35-44).

#### 2.3. Administrative Gatekeeping

 Administrative gatekeeping within primary care is a known barrier,^27,28^ where access to care is denied or delayed at reception or administrative stages. In lockdown, this most often occurred when patients phoned for appointments, or at the time of triage, but occurred on occasion in-person with acute issues (eg, an elderly man reportedly bleeding from a head injury, being told he needed an appointment through a closed glass door that he couldn’t hear through (S: M, 65+)). People did not like being triaged by reception, were reluctant to discuss private health concerns with a non-health-professional, and were not confident in advice transmitted through receptionists.

*“Did not appreciate being questioned by the receptionist as to why I needed to see the nurse, from a two metre distance in an area with other people” *(S: F, 55-64).

#### 2.4. Staff Manner

 The approach taken by practice staff (whether by phone, online or in-person) exacerbated or attenuated patient anxiety during lockdown, with a flow-on impact on healthcare access. Patients described how the way they were spoken to, and overall demeanour, of reception staff and clinicians could either calm or reinforce their fear, and directly influence their decision about whether to seek care. People understood why health professionals might be alarmed by COVID-19 and want to limit contact with patients, but could be put off when this alarm was overly apparent.

*“When I visited my medical centre, I felt very much at ease and calm. I didn’t know what to expect going in, but the staff I saw all appeared happy and were friendly. No one seemed stressed or overworked” *(S: F, 25-34).

*“They [general practice clinic staff] were sending out a lot of mixed messages about…the whole COVID thing…they were really, really scared, and that came across to us, as patients, that they were unsure of what to do” (I: F, 45-54).*

#### 2.5. Safety of the Health Service Environment

 For those who had an in-person consultation, changes to safety procedures (eg, PPE, hand sanitising, physical distancing) were noticed by patients immediately. Changes included having consultations, vaccinations or COVID-19 testing through a car window by staff wearing PPE; waiting outside the practice before being called in; or not being physically examined. These could have a positive impact on patients, by increasing their confidence in accessing healthcare safely, or a negative impact, if the experience was overly onerous or frightening.

*“The car park flu shot [and wait a short while after in the car park] made me feel safer than if I had to enter the clinic building” *(S: F, 45-54).

 Safety procedures could be insufficient to allay concerns if not well-executed; for example, if patients had to wait in what they perceived as confined areas alongside other patients. Although people accepted the need for safety, changes could be disconcerting. One interviewee’s experience of having a COVID-19 swab exemplified the worst aspects of this. She described driving into ‘an army-type environment, where you’ve got this makeshift tent and … these weird fences,’ with rows of cars filled with people who may be infectious (‘all you can hear them doing is coughing’). Health professionals in PPE asked questions without considering privacy (‘they’re yelling at them [in the cars] so you know what everyone’s temperature is’), forbade eye contact (“every time I went to look at them, they would say ‘don’t look at us!’”) and minimised any sense of personal connection (‘I couldn’t see their faces, I couldn’t see who they were so I had no idea, or no relationship with them’). Finally, the detritus from the swab was discarded into the car (‘instead of them passing you things, they just throw them into the car because they’re not wanting to touch you, because they’re not wanting to waste their PPE or whatever’) (I: F, 45-54). Although the interviewee understood the need for precautions, she was unprepared for how de-personalising and disrespectful the experience would feel.

####  3. Individual Influences on Healthcare Access

 Respondents’ decisions around seeking healthcare also reflected their own resources and ability to weigh up risks and benefits.

####  3.1. Self-management and Self-triage

 Many people chose to self-manage their health concerns when they were able to, particularly for health conditions that they were familiar with, either pre-existing, recurrent, or common. For newconditions, they considered what the condition might be, the severity of the symptoms and the consequences of not being immediately assessed. They made judgments about whether the condition might improve on its own and whether they could use over-the-counter medicines or other available treatments. Some monitored the symptoms and waited until later in lockdown before seeking treatment if the condition had not resolved. People also put on hold issues that they knew required a physical examination, and avoided seeking help for non-urgent problems, in response to perceptions related to public messages (see section 1.1) and the health service focus on COVID-19 and urgent issues only (see section 1.2).

*“For the gastric issue I waited four weeks trying to figure out what was going on, and going to the doctor was sort of the last resort when the pain became quite unbearable … I think under normal circumstances I would have definitely gone and seen [doctor] partway through that” *(I: F, 45-54).

#### 3.2. Conscious of Pressure on Health Services

 Respondents did not want to unnecessarily burden the health system. The motivation to undertake self-triage (see section 3.1) and delay seeking care was driven by concern that the public health system may be overwhelmed. This was particularly pressing in the first 2 weeks of lockdown when COVID-19 cases were proliferating, and it was uncertain whether lockdown would work. Patients worried that seeking help might cause stress to what was initially perceived to be a stretched health service. They also considered the needs of others, imagining that more serious issues would take priority, and acted on altruistic notions that other people’s problems, or COVID-19 infections, were more important.

*“I would’ve gone to the doctors if we weren’t in lockdown. And yes, I heard Ashley Bloomfield [Director-General of Health] say many times to seek help if you need it, but I couldn’t convince myself that my issues were more important than others”* (S: F, 25-34).

 When patients did seek healthcare, they recognised that clinicians were under stress and conveyed tolerance, often qualifying descriptions of mistakes with an explanation of why this may have occurred or an expression of understanding.

*“Given how stressed everyone is as humans and health professionals … everyone has done amazing. Someone forgot to ring me for my scheduled appointment but we are human” *(S: F, 45-54).

 However, sometimes service disruptions meant that people were put off trying to seek healthcare or they tried but were discouraged and gave up.

#### 3.3. OK/Not OK to Seek Routine Healthcare

 The health services’ focus on COVID-19 and urgent care (see section 1.2) directly affected patients who engaged with services, but also influenced those who may have wanted to seek healthcare. People felt the onus was on themselves to determine whether their health need was sufficiently important, which could lead to potentially urgent symptoms being underplayed or unrecognised.

*“A few times we’ll even have like Dr Bloomfield say, yeah, you should go to your doctor if you think you need to. But it’s that ‘if you think you need to’ … everyone feels that it’s a big scary pandemic out there and so maybe the slight pain in my heart doesn’t mean [it’s] serious”* (I: F, 25-34).

#### 3.4. Fear of Infection

 Patient fears about catching COVID-19 in a healthcare service could be allayed through a safe practice environment (see section 2.5), but patients were still justifiably worried about the risks of infection for themselves and others in their household. Waiting areas at general practices and hospitals were widely perceived to be one of the most likely places to be exposed to COVID-19, supported by reports of outbreaks in health settings in Europe.^4^

*“I was worried about going to a clinic with sick people there in case I picked up coronavirus” *(S: F, 55-64).

## Discussion

 From this study of the first 2020 COVID-19 lockdown in Aotearoa/New Zealand, just over half of respondents delayed seeking healthcare. At a national level, Government advice about accessing health services were perceived by some to conflict with messages about COVID-19 and lockdown restrictions, creating confusion about the appropriate management of non-COVID health problems. Changes in messaging were inevitable because of the uncertainty and evolving nature of the pandemic. What was needed, and what happened over time, was repeated clarifications of these changes. Stronger involvement of consumer groups in developing COVID-19 communications may also help mitigate such confusion.^29^ Unnecessary delays in seeking healthcare have been recognised as an unintended consequence of stay-at-home mandates and prioritisation of urgent healthcare.^13^

 Other health system factors were also at play. From a patient’s perspective, lockdown highlighted when health services functioned well, but also where services lacked integration, which was problematic when they needed quickly (eg, palliative care, some repeat prescriptions). Integration of services throughout Aotearoa/New Zealand is variable and difficult to assess,^30^ but improvements in the interfaces between secondary care, general practices and community pharmacies will be important for future extended lockdowns. 

 Some patients experienced delays because health services were unavailable or inaccessible, not through their choice. Early indicators from Aotearoa/New Zealand suggest that total deaths during this lockdown were lower than the same time period in previous years.^31^ However, the long-term impact of delays in cancer screening, routine investigations, and elective surgery are yet to be assessed. At a national level, a clear framework for how to prioritise screening and non-emergency procedures in lockdown situations would help both patients and health professionals navigate the uncertainty of postponing healthcare in a pandemic.^32^ This type of framework would have to contain guidance on weighing up the risks and benefits for individuals depending on how seriously the health system was affected, and how much of the health system needed to be reserved in case of an overwhelming outbreak.

 At a practice level, barriers to access included administrative gatekeeping and overtly stressed staff. Although patients understood why staff would feel anxious, this, along with a belief that services were stretched or would not deal with non-urgent issues, led to delays in seeking care. In a pandemic, fear and uncertainty predominate and affect both the general public and health professionals,^33,34^ but fear of infection could be reduced by obvious hygiene procedures and a calm, reassuring manner. Gatekeeping could be minimised with upskilling of reception staff and ensuring that clinicians answer health-related queries.

 From this research, those who delayed seeking care were more likely to be struggling financially, be concerned about the impact of COVID-19, have poorer health, a disability or more difficulties in managing their health in the pandemic. Proactive, up-to-date communication from practices about what services were available, how to get in touch and expected changes to service delivery could encourage access to services but needed to reach those most at risk. Practices with systems already in place to undertake electronic (website/portal/messaging), patient-focused communication were better able to provide care during lockdown, as has been found elsewhere.^35^ Outreach by non-digital methods can ensure that patients without online capability are not excluded.

 As well as health system and practice-level influences, individual patients weighed up the need to seek healthcare through self-assessment of their own illnesses. They considered the risk of COVID-19 exposure to themselves and others, and managed non-urgent problems by watching and waiting and self-care. Health systems, including Healthline and general practices, could provide more support for self-triage^36^ and routinely promote trusted patient-information sites, to help patients make appropriate decisions around how and when to access healthcare.

 Limitations of this research include that respondents were not fully representative of the overall population, with more female and European respondents. We cannot tell why people did not respond to the survey; perhaps they were unaware of they it, they did not think that the survey applied to them or they were unwilling to complete it. However, it could be the case that some groups had much lower access to healthcare than others; for example, the extreme disparity between genders suggests some level of inequity at play. Future research needs to be conducted to explore how pandemic lockdowns further exacerbate inequities in accessing to primary healthcare.

 The online nature of the survey meant that those without internet access or digital skills were less able to participate, and respondents were likely to be more comfortable with digital technology. As a result, we may not have identified how those in disadvantaged communities experienced delays in seeking healthcare, particularly those not enrolled in general practice. However, the online survey approach was the only practicable method during lockdown, given that in-person recruitment was prohibited, and seeking assistance from stressed general practices for recruitment was considered inappropriate.

## Conclusion

 In future lockdowns or crises, appropriate access to primary care could be improved with timely communication from practices to patients, proactive outreach, maintaining a physically safe and emotionally supportive practice environment and improving the integration between primary care and other health services. More support for patients to self-manage could benefit over-burdened health systems both in lockdown and in less extraordinary times.

## Acknowledgements

 The authors would like to thank all survey respondents and interviewees. Other members of the Primary Care Programme team were involved in securing funding for and the establishment of the programme.

 We thank our wider team members, professional and personal networks, colleagues known and unknown who helped in the dissemination of the online survey. We also want to acknowledge Dr. Lynne Russell’s (LR), Dr. Janet McDonald’s (JM), and Dr. Marianna Churchward’s (MC)assistance with interviews.

## Ethical issues

 This research was approved by the Human Ethics Committee of Victoria University of Wellington (ref #0000028485).

## Competing interests

 Authors declare that they have no competing interests.

## Authors’ contributions

 FI conceived of the study with approval from JC and KMH. FI led the survey development and creation, EM led development of the interview schedule and the rest of the team reviewed and approved these. Three of the authors were involved in interviewing (EM, FI, and JK) plus other researchers from the wider research team (LR, MC, and JM). MP led the analysis of the survey quantitative data. Coding and analysis of the open-ended survey questions was led by LM, with coding input and review by FI. FI led the analysis of the interview data, with coding input and review by EM, LR, MC, and KMH. FI drafted the manuscript and all authors read, revised and approved the final manuscript.

## Funding

 This research was funded by the New Zealand Health Research Council (ref # 18/667).

## Supplementary files

Supplementary file 1. Interview Schedule.

Supplementary file 2 contains Table S1.
