# Supplementary material for: Seeking Healthcare During Lockdown: Challenges, Opportunities and Lessons for the Future
Source: Int J Health Policy Manag. 2021 Apr 13;11(8):1316–24. doi: 10.34172/ijhpm.2021.26 (PMC9808356; doi:10.34172/ijhpm.2021.26)
Supplement: Supplementary file 1 — Interview Schedule. [file ijhpm-11-1316-s001.pdf]

Article title: Seeking Healthcare During Lockdown: Challenges, Opportunities and Lessons for the Future

Journal name: International Journal of Health Policy and Management (IJHPM)

Authors' information: Fiona Imlach[1], Eileen McKinlay[2], Jonathan Kennedy[2], Megan Pledger[1], Lesley Middleton[1], Jacqueline Cumming[1], Karen McBride-Henry[3]*

[1]Health Services Research Centre, Victoria University of Wellington, Wellington, New Zealand.

[2]Department of Primary Health Care and General Practice, University of Otago, Wellington, New Zealand.

[3]School of Nursing, Midwifery and Health Practice, Victoria University of Wellington, Wellington, New Zealand.

(*corresponding author: karen.mcbride-henry@vuw.ac.nz)

Supplementary file 1: Interview Schedule

Patient experiences of primary health care in a pandemic

Thank you again for agreeing to be interviewed for this research which focuses on people's experiences of general practice care during the COVID-19 pandemic lockdown period.
Living in general has been very difficult for many during the pandemic and I want you to know that we can stop this interview at any point if you are feeling uncomfortable for any reason. Let me know if you need to take a break.

Do you have any questions about the research before I begin?

First some general questions about yourself and your wellbeing and health during COVID-19.

1) Who was in your lockdown bubble?
2) What has mattered most to you about your health and wellbeing during this period?
3) Did you get COVID-19? Tell me about this?
4) How concerned are you about the impact of COVID-19 on your health?

Now, some questions about the (general practice or other term) you go to.

5) What is the general practice or health centre or clinic that you regularly attend? Do you have a doctor there that you usually see?
6) Do you know if [this practice] call themselves a Health Care Home?

Moving onto some questions about your health care during the COVID-19 pandemic.

7) During the COVID-19 pandemic lockdown, did you have to seek health care or advice about your health? Tell us about this. Some prompts:
   a) Who did you seek advice or care from?
   b) How long did it take to get a response?
   c) Generally speaking, what did you seek advice or care about? (e.g. COVID, screening, acute illness, chronic illness, care for another person, immunisation)
   d) Did you contact your regular general practice or another service? Did you see or talk to a clinician you already knew or someone unfamiliar? How important to you was contacting someone you knew in this circumstance?
   e) What type of contact did you have e.g. a physical visit/phone call/video call/e-portal consultation/COVID-19 station or something else
   f) Was the method of contact new to you? (phone/video/e-portal/COVID-19 station/Other)? How did you find this? Would you like to use this method when things settle down?
   g) How did you feel about the interaction – what was good about it or not so good?
   h) What was the outcome of the interaction, did you get what you needed?
   i) Did you pay for the care and information? How much was it (if you want to tell us that) – or was this more or less than a usual consult? Was cost a barrier to seeking care and information?
8) During the COVID-19 pandemic lockdown, did you delay or put off getting help for a health issue that you would have got help for under usual circumstances? Did a health care provider delay or put off care that you would normally have got (e.g. elective surgery, a lab test or scan, immunisation). Tell us about this. Some prompts:
   a) What did you/the health provider delay or put off?
   b) Why did you/the health provider delay or put this off?
   c) When will you get this health issue addressed?
   d) How does this delay make you feel?

Questions about alternative methods to face-to-face visits

During the lockdown, doctors and nurses in the community were told to provide alternatives to face-to-face visits whenever possible.

9) Thinking about telephone consults,
   i. In what situations would you prefer to talk to the doctor or nurse on the phone about a health issue, rather than have a face-to-face visit? Why?
   ii. In what situations would you prefer a face-to-face visit to a telephone consult? Why?
10) Thinking about virtual/video consults,
   iii. In what situations would you prefer to have a video call with the doctor or nurse on about a health issue, rather than have a face-to-face visit? Why?
   iv. In what situations would you prefer a face-to-face visit to a video call? Why?

Questions for those with long-term conditions

11) We know that people with chronic or long-term health condition often experience some difficulties in daily life and may need to visit their general practice more often. Do you have one or more chronic health conditions? (prompt if needed, e.g. heart disease, diabetes, high blood pressure, depression, anxiety, gout, arthritis, asthma, respiratory disease, etc). If yes, explore with the following prompts. If no, go to the next question.
   e) Before COVID-19, what role did your general practice play in helping you manage your health?
   f) Did this/has this changed during the COVID-19 pandemic? Prompts:
      i. Did staff from your regular general practice contact you to check on how you were getting along?

ii. Were you able to get a flu vaccination if you wanted one? How did this work for you?
iii. How did things go with getting repeats of your medicines?
iv. Were you able to go to the pharmacy to pick these up?
v. Do you normally get care from a physiotherapist or other health or social care professional? If yes, how did you access this during the pandemic?

g) During the COVID-19 pandemic did you make any changes to how you usually manage your health or care for yourself? If yes, ask what changes were made and why and what differences these have made and if the person will continue them. Prompts (if not mentioned): ask about diet, exercise, medicines, mental health, household tasks, shopping, isolation

Final questions

12) Is there anything else you want to tell us about your health and health care during the pandemic? Is there anything that could have been done differently to make your experience of health care better?

Demographics

Can I ask you a few questions about yourself.

13) Which age group do you belong to:
    - 18-24
    - 25-34
    - 35-44
    - 45-54
    - 55-64
    - 65-74
    - 75-84
    - 85 or older

14) Can you tell me which ethnic group or groups you belong to?
    - New Zealand European
    - Māori
    - Samoan
    - Cook Islands Māori
    - Tongan
    - Niuean
    - Chinese
    - Indian
    - Another ethnic group such as Dutch, Japanese or Tokelauan? Please say what it is____

15) Which of these statements best describes your **current** work situation (during the lockdown):
    - Working in paid employment (includes self-employment)
    - Not in paid work and looking for a job
    - Not in paid work and not looking for a job (for any reason, such as being retired, a caregiver, a homemaker, a full-time student)
    - Other (please specify)

16) Where do you live?
    - Northland
    - Auckland
    - Waikato
    - Bay of Plenty
    - Gisborne
    - Hawke’s Bay
    - Taranaki

- Manawatu-Whanganui
- Wellington-Wairarapa
- Tasman
- Nelson
- Marlborough
- West Coast
- Canterbury
- Otago
- Southland
- Other (specify)

17) Which of these best describes where you live?
    - A rural area
    - A small town (under 30,000 people)
    - A medium-sized town or large city major city (more than 30,000 people)
    - Other (specify)

If not already evident from the interview, ask

18) What is your gender?
    - Male
    - Female
    - Gender diverse
    - Prefer not to say

Support resources if patients become distressed

Healthline 0800 611 116
COVID Healthline 0800 358 5453
General mental health support
https://www.depression.org.nz/
https://www.allright.org.nz/
https://www.thelowdown.co.nz/
https://www.auntydee.co.nz/tips-and-help (mental health resource for Pasifika)
