# Supplementary material for: Seeking Healthcare During Lockdown: Challenges, Opportunities and Lessons for the Future
Source: Int J Health Policy Manag. 2021 Apr 13;11(8):1316–24. doi: 10.34172/ijhpm.2021.26 (PMC9808356; doi:10.34172/ijhpm.2021.26)
Supplement: Supplementary file 2 — contains Table S1. [file ijhpm-11-1316-s002.pdf]

Article title: Seeking Healthcare During Lockdown: Challenges, Opportunities and Lessons for the Future

Journal name: International Journal of Health Policy and Management (IJHPM)

Authors' information: Fiona Imlach[1], Eileen McKinlay[2], Jonathan Kennedy[2], Megan Pledger[1], Lesley Middleton[1], Jacqueline Cumming[1], Karen McBride-Henry[3]*

[1]Health Services Research Centre, Victoria University of Wellington, Wellington, New Zealand.

[2]Department of Primary Health Care and General Practice, University of Otago, Wellington, New Zealand.

[3]School of Nursing, Midwifery and Health Practice, Victoria University of Wellington, Wellington, New Zealand.

(*corresponding author: karen.mcbride-henry@vuw.ac.nz)

Supplementary file 2.

Table S1. Demographics of survey respondents and interviewees

**Characteristic**	**Survey n (%) Total n=1,010**	**Interviewees n (%) Total n=38**
**Age**		
18-34	221 (22)	7 (18)
35-44	201 (20)	6 (16)
45-54	247 (25)	12 (32)
55-64	173 (18)	3 (8)
65+	145 (15)	10 (26)
**Gender**		
Female	840 (85)	24 (63)
Male	141 (14)	14 (37)
Other[2]	13 (1)	-
**Prioritised ethnicity (in order of priority)**		
Māori	101 (10)	6 (16)
Pacific peoples	18 (2)	3 (8)
Asian	34 (3)	4 (11)
New Zealand European/Other	833 (85)	25 (66)
**Current work status**		

In paid employment as before COVID-19	581 (59)	22 (58)
In paid employment with reduced pay due to COVID-19	108 (11)	3 (8)
In paid employment but not being paid due to COVID-19	26 (3)	-
Unemployed and looking for a job	31 (3)	-
Not in paid employment and not looking for a job	240 (24)	13 (34)
**Grouped District Health Board (DHB) areas**[3]		
Upper North Island	205 (21)	7 (18)
Central North Island	118 (12)	3 (8)
Lower North Island	437 (44)	20 (53)
South Island	232 (23)	8 (21)
1: The maximum margin of error for a simple random sample of size 1010 is 3.1% 2:Those who answered "gender diverse" or "prefer not to say" were grouped together because of small numbers; 3:Upper North Island = Northland, Waitematā, Auckland and Counties Manukau DHBs; Central North Island = Waikato, Bay of Plenty, Tairāwhiti, Lakes, Taranaki DHBs; Lower North Island = Whanganui, Hawke's Bay, MidCentral, Wairarapa, Hutt, Capital and Coast DHBs; South Island = Nelson-Marlborough, West Coast, Canterbury, South Canterbury, Southern DHBs		
